# The Phylogeny of *Rickettsia* Using Different Evolutionary Signatures: How Tree-Like is Bacterial Evolution?

**DOI:** 10.1093/sysbio/syv084

**Published:** 2015-11-11

**Authors:** Gemma G. R. Murray, Lucy A. Weinert, Emma L. Rhule, John J. Welch

**Affiliations:** ^1^Department of Genetics, University of Cambridge, Downing Street, Cambridge CB2 3EH, UK; and; ^2^Department of Veterinary Medicine, University of Cambridge, Madingley Road, Cambridge CB3 0ES, UK

**Keywords:** Gene tree discordance, genome reduction, horizontal gene transfer, systematic error, reticulate evolution

## Abstract

*Rickettsia* is a genus of intracellular bacteria whose hosts and transmission strategies are both impressively diverse, and this is reflected in a highly dynamic genome. Some previous studies have described the evolutionary history of *Rickettsia* as non-tree-like, due to incongruity between phylogenetic reconstructions using different portions of the genome. Here, we reconstruct the *Rickettsia* phylogeny using whole-genome data, including two new genomes from previously unsampled host groups. We find that a single topology, which is supported by multiple sources of phylogenetic signal, well describes the evolutionary history of the core genome. We do observe extensive incongruence between individual gene trees, but analyses of simulations over a single topology and interspersed partitions of sites show that this is more plausibly attributed to systematic error than to horizontal gene transfer. Some conflicting placements also result from phylogenetic analyses of accessory genome content (i.e., gene presence/absence), but we argue that these are also due to systematic error, stemming from convergent genome reduction, which cannot be accommodated by existing phylogenetic methods. Our results show that, even within a single genus, tests for gene exchange based on phylogenetic incongruence may be susceptible to false positives.

*Rickettsia* is a genus of intracellular symbionts that exemplifies the extraordinary adaptive potential of bacteria (Perlman et al. 2006; Weinert et al. 2009a; Gottlieb et al. 2011; Weinert 2015). Many *Rickettsia* infect arthropods, but the host range of the genus also includes protozoa, algae, plants, and vertebrates. Its adaptations to these hosts are equally diverse, and encompass an array of mutualistic and parasitic interactions, including a number of reproductive manipulations. The genus is, however, best known for causing acute human diseases, such as typhus and Rocky Mountain spotted fever (Raoult and Roux 1997; Parola et al. 2005).

A robust phylogeny is essential for understanding the adaptive radiation of the *Rickettsia* genus. Several previous studies have used molecular data to estimate this phylogeny (including Sekeyova et al. 2001; Vitorino et al. 2007; Gillespie et al. 2008, 2012, 2014a; Merhej et al. 2009a, 2014; Weinert et al. 2009a; Georgiades et al. 2011; Merhej and Raoult 2011; Driscoll et al. 2013), and while these reconstructions agree in several respects, some placements differ. One possible reason for these disagreements is the non-tree-like or reticulate nature of bacterial evolution, and it is clear that *Rickettsia* can acquire genes through horizontal transfer. For example, some *Rickettsia* possess conjugative plasmids, or encode conjugation genes in their genomes, and a number of horizontally acquired genes have been identified (e.g. Blanc et al. 2007b; Gillespie et al. 2007, 2012, 2014a; Weinert et al. 2009b). Moreover, *Rickettsia* genomes contain an unusually large proportion of degraded genes in the form of pseudogenes or “junk” DNA (Andersson and Andersson 1999, 2001), and they greatly vary in size and gene content. This is likely to reflect not only varying degrees of genome reduction, which is believed to be ongoing in *Rickettsia* (Andersson and Andersson 1999 2001; Ogata et al. 2001; Blanc et al. 2007a), but may also reflect horizontal gene transfer (Wolf et al. 1999; Blanc et al. 2007b; Gillespie et al. 2007, 2012, 2014a; Fuxelius et al. 2008; Georgiades et al. 2011; Merhej et al. 2011; Le et al. 2012; Hernandez-Lopez et al. 2013). However, the dynamic nature of *Rickettsia* genome evolution could also lead to a high level of systematic error in phylogenetic reconstructions, meaning that discordant phylogenies need not reflect a genuine plurality of evolutionary histories (Betancur-R. et al. 2013; Sharma et al. 2014).

This study presents a comprehensive analysis of *Rickettsia* phylogeny, using whole-genome data. In addition to published sequences, we present two new genomes of special interest. The first comes from the symbiont of the ladybird beetle *Adalia bipunctata*, and is the first genome of a *Rickettsia* known to persist as a male-killer (Werren et al. 1994; Hurst et al. 1999; Majerus et al. 1999). The second comes from the symbiont of the ciliate protozoan that infects freshwater fish, *Ichthyophthirius* *multifiliis* (data from Sun et al. 2009), and is the first sequenced genome of a *Rickettsia* without an arthropod host.

Our study uses several approaches to identify and correct for systematic error in the reconstructions of the *Rickettsia* phylogeny, and to test for evidence of horizontal gene transfer. These include the use of non-stationary models, comparison of multiple quasi-independent sources of phylogenetic signal, and comparison with simulations over known topologies. Taken together, our results suggest that much of the apparent evidence for reticulate evolution in the *Rickettsia* core genome, and in patterns of gene presence/absence, stems from systematic error, sometimes resulting from evolutionary processes that are not well described by any existing phylogenetic model. After accounting for this error, we argue that the evolution of *Rickettsia* is largely described by a single topology.

## Methods

### *Sequencing the* R. *symbiont of* A. bipunctata

*Adalia bipunctata* infected with a male-killing *Rickettsia* were obtained from the Cambridge area in 2011. *Rickettsia* cannot be cultured easily, so we extracted DNA from both ovarian tissue (extracted under a dissecting microscope) and unfertilized (haploid) eggs from a single individual. We ground these tissues with a sterile micropestle in a microcentrifuge tube and extracted the DNA using the QIAamp micro DNA Micro kit according to the manufacturer's instructions (Qiagen, Manchester, UK). To quantify the relative amounts of *Rickettsia* and ladybird beetle DNA, we designed new primers to amplify approximately 100 bp of the *gltA* and *atpA* genes of previously sequenced *Rickettsia* from *A. bipunctata* (Weinert et al. 2009a), and the ladybird beetle *g6pd* gene (see Supplementary Table S2 available on Dryad at http://dx.doi.org/10.5061/dryad.6cn66). We used these primers with a serial dilution of DNA to produce a standard curve using qPCR. For these gene products in these DNA extractions, ovarian tissue contained around twice as much *Rickettsia* DNA as did comparable amounts of egg tissue. However, ovarian tissue also contained much more host DNA (the ratio of *Rickettsia* DNA to ladybird DNA was ∼8 in ovaries, as opposed to ∼4000 in eggs). For this reason, we retained the DNA from the eggs for genome sequencing. To increase the yield of extracted DNA for genome sequencing, the DNA was treated with an illustra GenomiPhi V2 DNA amplification kit according to manufacturer's instructions (GE Healthcare Life Sciences, Buckingham, UK). The amplified samples were cleaned prior to sequencing with a QIAamp DNA mini kit according to manufacturer's instructions (Qiagen, supplementary protocol for Purification of REPLI-g amplified DNA). The amplified DNA was used for the construction of one 3 kb paired-end 454 library that was sequenced on 1/12 plate of a 454 Roche FLX machine (Biochemistry department, Cambridge, UK) and one paired-end Illumina library (TruSeq kit) that was sequenced on 1/12 lane of a HiSeq 2000 (TGAC, Norwich). Roche's *Newbler* assembly software was used under the default parameters to perform a *de novo* assembly. Host contamination was identified through nucleotide BLAST searches (BLASTN) of the assembled scaffolds against GenBank (http://www.ncbi.nlm.nih.gov/genbank/). Scaffolds that were most similar to sequences from beetles were removed from the assembly.

### Collecting the Other Genomes

All available *Rickettsia* whole-genome sequences were obtained from GenBank (http://www.ncbi.nlm.nih.gov/genbank/; accessed 2012) (Supplementary Table S1). The unpublished genome of the *Rickettsia* that infects *Ichthyophthirius multifiliis* was kindly provided by Prof. R. S. Coyne, Dr T. Doak, and Prof. M. Lynch. This genome was obtained as a result of sequencing its host, as described by (Sun et al., 2009).

### Identifying Homology Groups

We first identified orthologous genes in the 50 existing *Rickettsia* genomes using *OrthoMCL* with the recommended inflation value of 1.5 (Li et al. 2003). As input, we used the annotations of the published genomes from the online PATRIC database (Wattam et al. 2014), and an annotation of the R. symbiont of *I. multifiliis* (Doak, T. and Lynch, M., 2013, personal communication). After automatic alignment with MACSE (Ranwez et al. 2011), orthology groups were manually curated (sequences from each group are provided in the Supplementary Information).

First, in order to identify potential pseudogenes, we defined as “truncated,” any gene that was <80% of the median length of its group (cf. Lerat and Ochman 2005; Ochman and Davalos 2006). Second, to identify clear cases of segregating paralogues wrongly placed within a single orthology group, we examined by eye all groups that contained multiple members from a single genome. Groups in which there was a clear division, best explained by divergence early in or prior to the diversification of *Rickettsia*, were re-described as multiple groups. Third, to identify orthologous genes that had been wrongly placed in distinct orthology groups (e.g., due to translation differences between annotations), we performed an all-against-all gene group nucleotide BLAST search (BLASTN) (Camacho et al. 2013). All hits were examined by eye. Groups that could be aligned with confidence were re-described as a single group, and cases in which the relationship between the groups could not be clearly established were excluded from subsequent analyses.

Next, we checked all cases of absent and truncated gene groups by performing a BLASTN search of all orthology groups against all the genomes in which they were described as absent or truncated. Genes were then re-described as present if there was a BLASTN hit for which (1) a start codon was present, (2) the hit region (before any stop codon) was >80% of the median length of that group, (3) a translation of the hit region had an identity to the top hit of the gene group of >65%, and (4) the gene group was the reciprocal best hit of the hit region. Genes were re-described as “truncated” if they failed to meet any of the criteria (1)–(4), but had a BLASTN hit that was >25% of the minimum length of non-truncated members of the group, and if groups members were the reciprocal best hit of the region. After completing orthology groups for the 50 existing genomes, to determine the gene content of the R. symbiont of *A. bipunctata*, we used a BLASTN search of all group alignments against the scaffolds of the assembled genome.

Finally, any orthology group that contained a member that was annotated as appearing on a plasmid was excluded from phylogenetic analyses, since the absence of plasmids cannot be inferred from their not being captured in sequencing (Baldridge et al. 2008).

### Scoring Characters in the Core Genome

Unique core genes, i.e., genes present once and only once in all strains, were identified in the orthology groups, aligned using *MACSE* (Ranwez et al. 2011), corrected by eye, and checked for potential translation (i.e., lack of premature stop codons or frame-shifting indels). Genes that were too divergent to align with confidence, or that were truncated in any completely assembled genome, were excluded from membership of the core genome, and so were small regions too divergent to align with confidence. SNPs and fixed sites were not scored for codons that were absent in more than one genome, since missing data can result in artifacts in phylogenetic reconstruction (Roure et al. 2013).

To score indels within the core genes, we excluded those found within 100 bases of gene boundaries, which might involve localized shifts in reading frame. This yielded 382 indels. We then excluded any indel whose boundaries overlapped with any of the other indels in the data set, since overlaps make it difficult to count events without ambiguity. This left us with the final data set of 240 non-overlapping indels used to estimate the topology. To score re-arrangements in the core genes, we used only the completely assembled genomes (Supplementary Table S1). Inversions and translocations were identified by hand, or using the double cut and join metric of Yancopoulos et al. (2005) as implemented in *UniMoG* (Hilker et al. 2012). For the phylogenetic analysis of gene presence/absence, we treated truncated genes (see above) as absent, but all analyses were repeated with these genes scored as present.

To identify “synteny blocks” of core genes, we first removed the few strains that had undergone high rates of genome rearrangement (the Bellii group, *R. peacockii*, andR. symbiont of *I. scapularis*; Supplementary Fig. S3a), and then identified 55 groups of contiguous core genes. These varied in size from 1 to 64 genes.

### Phylogenetic Analyses

Our phylogenetic analyses were carried out using Bayesian inference. Convergence (stationarity of traces and effective samples sizes >200) was assessed using *Tracer* v1.6 and burn-in removed as required for each run (Rambaut and Drummond 2013). To implement stationary models of evolution, we used *MrBayes* v3 (Huelsenbeck and Ronquist 2001). In a Bayesian framework, over-parameterization does not generally result in poor performance (since unidentifiable parameters will just revert to their priors) (Holder and Lewis 2003), so we chose the largest of the standard substitution models consistent with the number of character states. For four-state analyses (i.e., ACGT-coded sequences), we used a GTR+Γ model. For two-state analyses (i.e., RY-coded SNPs, indels, and gene presence/absence), we used a two-state analogue of the F81+Γ model. This was implemented using a “restriction model” in *MrBayes* or by using a non-standard two-character alphabet in *Phylobayes* v3.3 (Lartillot et al. 2009). When all three codon positions were included in a single analysis, we treated third positions as a separate partition with its own relative rate of evolution. When fixed sites were excluded *a priori* (e.g., for indels and gene presence/absence), we used the “restriction model” (Huelsenbeck and Ronquist 2001) to correct for this ascertainment bias.

**Figure 1.**
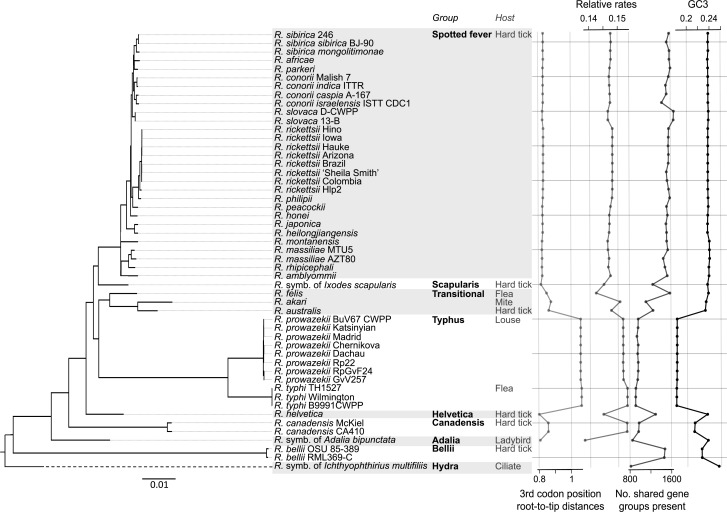
*Rickettsia* phylogeny estimated from 1st and 2nd codon positions in the concatenated core genes. Forty-six out of 48 nodes have posterior probabilities of 100%, and the two remaining nodes with <50% support are collapsed. The tree is midpoint rooted. The membership of previously described *Rickettsia* groups and the host groups that these strains infect are described (cf. Weinert 2015). Plots show (1) the root-to-tip distance (substitutions per site) in the tree estimated from 3^rd^ codon positions, (2) the relative rate of amino acid changing substitutions, as estimated from the ratio of root-to-tip distances at 1st and 2nd codon positions, relative to that at 3rd codon positions, (3) the number of shared gene groups present in each strain (scoring truncated genes, likely to be pseudogenes, as “absent”), and (4) the GC content of 3rd codon positions in the core genome.

To apply non-stationary models of evolution we used *Phylobayes* v3.3 (Lartillot et al. 2009). This method requires the specification of an outgroup. The *Rickettsia bellii* strains were used for this purpose, since they are consistently placed as an outgroup both in our analyses (e.g., Fig. 1 and Supplementary Fig. 1) and in published studies of *Rickettsia* phylogeny (including Gillespie et al. 2008, 2012, 2014a; Merhej et al. 2009a, 2014; Weinert et al. 2009a; Merhej and Raoult 2011; Driscoll et al. 2013). Rates and character frequencies were allowed to vary over lineages, and for analysis of SNPs, to aid convergence, we modeled the between-site rate variation with an empirical mixture of profiles taken from the “C20” model (Quang et al. 2008).

### Testing for Systematic Error in the Genome Tree

To test whether our estimate of genome-wide topology from SNPs in the core genes was affected by heterogeneity in the evolutionary process, we re-estimated the topology from various subsets of the data. In particular, we (1) removed transition substitutions, by recoding each base as either purine or pyrimidine (RY coding) (see Phillips 2009; Lanfear et al. 2010); and (2) removed rapidly evolving sites, which were defined as any site that varied within well-sampled, closely related clades, namely the Spotted fever group, the *R. prowazekii* strains, the *R. typhi* strains, and the *R. canadensis* strains (Fig. 1). These analyses aimed to remove long-branch artifacts due to sequence saturation, and artifacts due to heterogeneity in GC content. However, we also observed variation in purine content that weakly correlates with variation in GC content when genes are aligned 5′-3′ (Supplementary Table S1), and so we (3) repeated the analysis of RY coded 3rd positions with a non-stationary model of purine content.

### Inference of Gene Exchange in the Core Genome

Many model-based methods are available for testing for a plurality of evolutionary histories among genes. For our *Rickettsia* data it was not computationally feasible to use an approach such as *GARD* (Kosakovsky Pond et al. 2006), which infers “breakpoints” dynamically across a single alignment. Methods such as *ClonalFrame* (Didelot and Falush 2007), which assume constant evolutionary rates (i.e., constant root-to-tip distances), are also clearly invalid for our data, given the high level of rate variation observed (Fig. 1). Both approaches are also difficult to interpret when there has been a high rate of genome rearrangement (see Supplementary Fig. S3a). Accordingly, we chose to estimate single-gene trees, and compare these to the genome-wide consensus. We also compared gene trees using a multiple co-inertia analysis (Dolédec and Chessel 1994; Chessel and Hanafi 1996; Hernandez-Lopez et al. 2013). This method simplifies the signal in the data by describing the incongruence between gene trees as a distance in a two-dimensional space, but it does provide a straightforward method of identifying strains and genes with a high level of incongruity (through calculating a score for each gene and strain). Multiple co-inertia analyses were implemented in *Phylo-MCOA* (de Vienne et al. 2012), using the 50% cutoff for the input trees, and a nodal measure of phylogenetic distance between strains (results were similar when a higher cutoff of 95% was used; see Supplementary Fig. S5).

### Evolutionary Simulations

**Figure 2.**
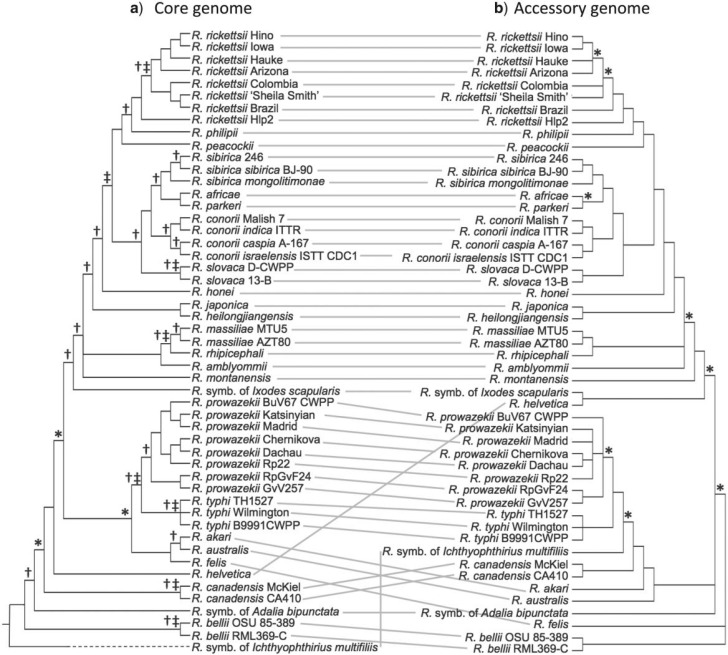
The topology of the *Rickettsia* phylogeny established (a) through analysis of SNPs in the core genome at 1st and 2nd codon positions and 3rd codon positions separately, and (b) through analysis of the presence and absence of accessory genes (scoring truncated genes, likely to be pseudogenes, as “absent”). Symbols on nodes show different sources and levels of support. a) * indicates nodes estimated with <95% support from 3rd codon positions (Supplementary Table S3); †>95% support from indels (Supplementary Fig. S2); ‡ supports from a genome rearrangement (Supplementary Fig. S3a). b) * indicates <95% support from presence/absence of accessory genes (Supplementary Fig. S4).

To simulate the evolution of the *Rickettsia* core genes over a single topology, we first used the real *Rickettsia* data, and the fixed topology estimated from the concatenated core genome (Fig. 2a), to estimate branch lengths and all parameters of a non-stationary F81+Γ model, with branch-specific base frequencies (Lartillot et al. 2009). Separate estimates were obtained for (1) the first two codon positions and (2) third codon positions, and each was obtained from a randomly chosen subsample of 10,000 such sites from the concatenated core genome alignment. We then used these parameters to simulate a complete core genome of 228,123 sites (including 76,041 third positions), using *INDELible* (Fletcher and Yang 2009). Finally, we split up this simulated core genome to form 458 simulated core genes, whose lengths matched those of the real genes. To reflect rate variation among the real genes, we first calculated an “entropy score” (Xia et al. 2003), for all sites in the real and simulated core genomes. We then chose sites from the simulated genome so that the rank order of entropy scores was identical between the real and simulated data sets (so, e.g., if a real gene contained the six third codon positions with the highest entropy scores in the real genome, the equivalent simulated gene would contain the six third codon positions with the highest entropy scores in the simulated genome). We chose to use entropy scores—rather than, say, rate estimates—because entropy is calculated from allele frequencies at a site, without making any assumptions about the topology over which the alleles evolved (Xia et al. 2003). This approach therefore avoids the potential circularity of simulating genes with high level of homoplasy, when the “homoplasy” in the equivalent real genes could have been due to a failure to account for real instances of gene exchange.

The simulations of gene presence/absence followed a similar procedure, but with simpler, two-state evolutionary models. Evolutionary parameters of stationary or non-stationary models were estimated from the real data, as described above, with the topology fixed to match Figure 2a. We then used *INDELible* to simulate the presence/absence of 1891 genes (matching the number of non-core genes in the real data set that were present in two or more strains). For each strain in each data set, we then calculated the number of genes present, denoted S, and the odds ratio, *OR*, which describes the similarity in gene content between each simulated genome and the smallest simulated genome, correcting for their sizes. This odds ratio is defined as *OR* = ($P_{11}/P_{10})$/($P_{01}$/$P_{00})$, where $P_{10}$ is the number of genes present in the smallest genome but absent in the current genome, $P_{01}$ is the number of genes present in the current genome but absent in the smallest genome, and $P_{11\;}(P_{00})$ is the number of genes that are present (absent) in both genomes. Using the same notation, the size of the current genome is $S=P_{01}+P_{11}$. When genomes are equally related to the smallest genome, it follows from these definitions that *OR* is not expected to correlate with S, unless there has been some degree of evolutionary convergence (i.e., replicated losses or gains).

**Figure 3.**
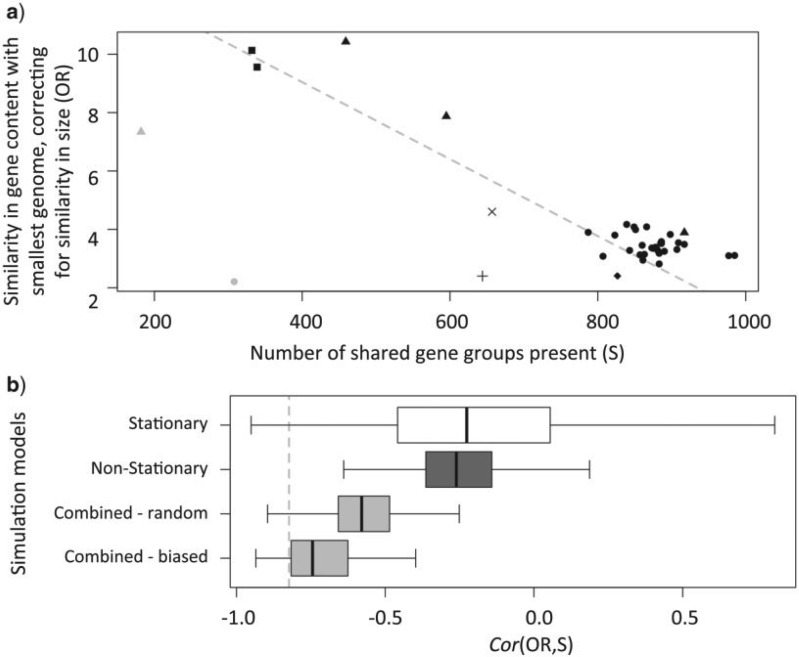
Evidence for convergence to a minimal core genome, leading to between-site variation in non-stationarity. a) The number of shared gene groups present (S; x-axis) plotted against the similarity in gene content with the smallest genome (*R. typhi* Wilmington), correcting for similarity in size (OR; y-axis) (see “Methods” section for details). Symbols indicate *Rickettsia* groups (shown in Fig. 1): •, Spotted fever; ▴, Transitional; ♦, Bellii; ▪ Canadensis; ×, Helvetica; +, Scapularis; 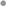, Adalia; and 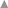, Hydra (other Typhus group strains are not shown due to their close relationship to the smallest genome). b) Correlation coefficients corresponding to the linear regression shown in (a) for sets of accessory genomes simulated under different evolutionary models. The gray dashed line shows the correlation coefficient of the real data. Boxes show results from 1000 simulated data sets, each simulated over the genome tree, under four different evolutionary models of gene gain/loss: a stationary model, a non-stationary model, a combination of the stationary and non-stationary model (where 50% of genes are described by each), and a combination of the stationary and non-stationary model (where 50% of genes are described by each, and where genes are more likely to be present under the stationary model and absent under the non-stationary model) (see “Methods” section).

To match the pattern observed in the real data (Fig. 3a), Figure 3b shows the correlations calculated from a subset of 10 simulated strains, one from each of the Spotted Fever group, *R. helvetica*, R. symbiont of *I. scapularis*, *R. felis*, *R. australis*, and *R. akari*, and one representative from each of *R. typhi*, *R. prowazekii*, *R. canadensis*, and *R. bellii*.

### Testing for “Internal Contradictions”

Intralocus recombination means that contiguous sets of sites in a gene might have evolved over different genealogies. Incongruent topologies can also arise from systematic error, but the sets of sites involved need not be contiguous. Indeed, systematic error can result in incongruent topologies from sets of sites that are physically interspersed, and so must have followed the same genealogy in reality. As such, to test for systematic error, we identified genes where interspersed sets of sites supported different topologies, while contiguous sets of sites supported the same genealogy. Such genes are said to show “internal contradictions,” and to show “strong internal contradictions” if the conflicting topologies each receive >95% posterior support. In detail, we re-estimated separate gene trees for (1) third codon positions and (2) the first two codon positions. When we found disagreement between these topologies, we carried out a further test to be sure that the conflicting signal could not be explained by intralocus recombination. For this purpose, for single genes we ran recombination tests using *GARD* on the *Datamonkey* webserver (Kosakovsky Pond et al. 2006; Delport et al. 2010). For the larger synteny blocks, it was not possible to run *GARD*, and so we examined the single-gene trees for genes within the block, to confirm that no two subsets of contiguous genes supported mutually contradictory placements.

### Comparing Apparent Evidence of Gene Exchange to Observed Levels of Systematic Error

To compare the overall evidence for gene exchange to the overall evidence for systematic error, we defined four quantities that could be calculated for each node in the *Rickettsia* genome tree. First, we define $p_{i,ge}$ as the proportion of strongly supported (>95%) contradictory nodes present in the real single-gene trees that contradict node i, and $p_{i,se}$ as the same quantity for the simulated genes (all of which were simulated over the genome tree, which contains node i). We defined similar quantities for the synteny block analyses. In particular, we defined as $n_{i,se}$ the number of synteny blocks where node i in the genome tree was contradicted with strong support by one partition, but where this contradictory placement was itself contradicted with strong support by the other partition. Analogously, $n_{i,ge}$ was defined as the number of synteny blocks where node i was contradicted with strong support by one partition, but where this contradictory placement was not contradicted with strong support by the other partition. These quantities were then normalized, such that $q_{i,se}=n_{i,se}/\sum_{j}n_{j,se}$ and $q_{i,ge}=n_{i,ge}/\sum_{j}n_{j,ge}$. The combined evidence for systematic error affecting a particular node is defined as the mean of $p_{i,se}$ and $q_{i,se}$, while the combined evidence for gene exchange affecting a particular node is defined as the mean of $p_{i,ge}$ and $q_{i,ge}$. Because these values are proportions, and many placements are entirely consistent across reconstructions (i.e., the values are zero for many nodes), we used a standard variable transformation to define two indices $I_{i,ge}=\text{arcsin}\left(\sqrt{\frac{1}{2}(p_{i,ge}+q_{i,ge})}\right)$, and $I_{i,se}=\text{arcsin}\left(\sqrt{\frac{1}{2}(p_{i,se}+q_{i,se})}\right)$ (Sokal and Rohlf 1981). A strong correlation between these indices indicates that nodes with high levels of apparent gene exchange are also subject to high levels of systematic error (Fig. 5).

### Presence of Conjugation Genes

To establish the presence/absence/truncation of conjugation genes in the *Rickettsia* genomes, we performed a protein-level BLAST search (BLASTP) of genes that were annotated as conjugation genes in the PATRIC database against our orthology groups (Wattam et al. 2014). We performed a protein level BLAST of these groups against the GenBank archive to check that they contained conserved domains consistent with their annotations (http://www.ncbi.nlm.nih.gov/genbank/; accessed April 2015). We visually inspected all alignments to check for segregating paralogues (which were described as copies of the same gene) and truncation.

To investigate the effects of conjugation genes on *Rickettsia* evolution, we used the number of non-truncated conjugation genes as a predictor in regression analyses. In all cases, we accounted for phylogenetic relatedness, using generalized least squares (GLS). In particular, we used the phylogeny of Figure 1, with Pagel's λ correlation structure (Pagel 1999; Freckleton et al. 2002), as implemented in the R packages *nlme* and *ape* (Paradis et al. 2004; Pinheiro et al. 2015). Standard transformations were used to standardize the variance (a square root for counts of conjugation genes, and a logarithm for genome size in bp). In all cases, λ estimates were close to unity, confirming the need for a phylogenetically corrected approach.

### 16S rRNA phylogeny

To place the R. symbiont of *I. multifiliis*, nucleotide sequences of 16S rRNA from our *Rickettsia* genomes were combined with sequences from distantly related *Rickettsia* and members of other Rickettsiales obtained from GenBank (http://www.ncbi.nlm.nih.gov/genbank/; accessed 2013). Sequences were aligned by eye and we removed any bases missing from more than one sequence (the alignment and accession numbers are provided as Supplementary Information). The phylogeny was estimated using the same method as was used for the core genes.

## Results

To investigate the phylogeny of *Rickettsia*, we analyzed the published genomes of 49 strains, representing 23 named species (Supplementary Table S1), and two newly sequenced strains from new host groups. The symbiont of the ladybird beetle *Adalia bipunctata* was assembled into 80 scaffolds (provided in the Supplementary Information). The large number of scaffolds was probably due to repetitive regions and/or artifacts of the DNA amplification process. The symbiont of the ciliate protozoan, *I. multifiliis*, was assembled into three contigs (Doak, T. and Lynch, M., 2013, personal communication). While both new genomes are incompletely assembled, 43/49 published genomes are complete.

### Phylogeny Estimated from the Core Genome

We first constructed a phylogeny of *Rickettsia*, its sister genus *Orientia*, and other more distant Rickettsiales, from the conserved 16S rRNA. This phylogeny placed the R. symbiont of *I. multifiliis* within the distantly related Hydra group of *Rickettsia* (Supplementary Fig. S1; Weinert et al. 2009a), while all of our other genomes were placed within a relatively closely related group of arthropod-associated strains. This was reflected in the analysis of gene content, with the R. symbiont of *I. multifiliis* lacking many shared genes, and containing many others that were not alignable with confidence. For this reason, we excluded the R. symbiont of *I. multifiliis* genome from our phylogenetic analysis of the *Rickettsia* core genome, and aligned a set of 458 unique core genes for the remaining 50 strains. Concatenating these alignments, we estimated the phylogeny from SNPs at third codon positions, and, independently, from the first two codon positions. The resulting phylogeny was well supported, consistent across codon positions (Figs. 1 and 2a), and with some recent studies (e.g., Driscoll et al. 2013; Gillespie et al. 2014a).

### Testing for Systematic Error

While the topology we have estimated is well supported, it might not reflect the true evolutionary history of *Rickettsia* due to systematic error and model inadequacy (Philippe 2000; Rodríguez-Ezpeleta et al. 2007; Driscoll et al. 2013). This possibility is raised by the large variation in *Rickettsia* GC content and evolutionary rate (Fig. 1), and by a previous study that showed that base composition bias affects topological inferences for other members of the Rickettsiales (Driscoll et al. 2013). We used two approaches to test the robustness of our phylogeny. First, we re-analyzed the core genome alignment allowing or correcting for variation in the evolutionary process (see ‘Methods’ section). In every case, the estimated topology agreed with Figure 2a (see Supplementary Table S3 for details).

A second approach to testing for systematic error is to use distinct and quasi-independent sources of phylogenetic signal. We first considered insertions and deletions (indels) in the core genes. In agreement with results from other bacteria (Williams and Wernegreen 2012), we found evidence of indel hot spots: many indels overlapped with each other, and 18/240 non-overlapping indels formed homoplasies when mapped to the topology of Figure 2a. Nevertheless, a phylogeny estimated from these indels alone provides support for many of the same groupings (marked as dagger in Fig. 2a), and no significant support for any incongruent grouping (Supplementary Fig. S2).

A second, quasi-independent source of phylogenetic signal is genomic rearrangements. These too occur at hot spots, with discontinuities in gene order often occurring at the same locations in our *Rickettsia* genomes (Supplementary Fig. S3b; Belda et al. 2005; Darling et al. 2008). Furthermore, many of the rearrangements map to terminal branches, which suggests that they probably represent deleterious mutations, unlikely to contribute to ongoing evolution (Parkhill et al. 2001; Rocha et al. 2006; Darling et al. 2008; Ho et al. 2011). Nevertheless, 12/33 inversions that we could reconstruct with confidence mapped onto internal branches of the *Rickettsia* phylogeny, where they provide support for the tree topology in Figure 2a (marked as double dagger; see also Supplementary Fig. S3a). There was only one case of homoplasy, with identical inversions mapping to three distinct terminal branches (*R. akari, R. massiliae* AZT80, and *R. amblyommii*) whose grouping found no support from any of our subsequent analyses.

### The Presence/Absence of Accessory Genes

We next inferred topology from the presence/absence of accessory genes (i.e., genes present in some, but not all of our *Rickettsia* strains). The resulting topology (Fig. 2b), is similar to the analysis of (Georgiades et al., 2011), and is mostly consistent with the core genome tree (Fig. 1). There are, however, some highly supported differences at basal nodes, but these unusual placements were not robust to minor changes in the data or model. For example, different sets of incongruent, but highly supported, basal relationships are obtained if we allow for non-stationarity in rates of gene gain and loss, if we code truncated genes as present rather than absent, or if we fail to correct the likelihood for *a priori* missing states (e.g., universally absent genes; see Supplementary Fig. S4).

These disagreements might indicate substantial reticulate evolution deep in the tree, since similarity in gene content between distantly related *Rickettsia* might indicate extensive gene exchange between them (Ochman et al. 2000; Gogarten et al. 2002). However, the placements could also reflect systematic error. The clearest difference between Figure 2a and 2b is that the smallest genomes cluster together in the phylogeny inferred from gene presence/absence (Supplementary Table S1). This is unlikely to be the result of gene exchange, because the Typhus and Canadensis groups, whose small genomes cluster in Figure 2b, have non-overlapping host ranges (Fig. 1), and there is evidence that they both have small effective population sizes, consistent with population isolation (Fig. 1 and Supplementary Table S1; Moran et al. 2009). Furthermore, replicated genome reduction may be associated with convergence to a similar minimal core genome (Moran 2003; Merhej et al. 2009b; McCutcheon and Moran 2012), so small genomes can have similar gene contents, even if they are distantly related and underwent no gene exchange.

A pattern of convergent genome reduction is evident in our *Rickettsia* data. Indeed, a simple correlation shows that the smaller *Rickettsia* genomes are closer in gene content than would be predicted, even after correcting for their size (Fig. 3a). The same pattern is present between the major groups, and within groups that vary greatly in genome size (e.g., the Transitional group; Fig. 3a). The only outliers are the two new genomes presented here, probably representing an underestimation of shared gene content in strains that are too divergent (R. symbiont of *I. multifiliis*) or poorly assembled (R. symbiont of *A. bipunctata*). The sort of evolutionary convergence suggested by Figure 3a cannot be accommodated by standard non-stationary processes, such as those used above to model variation in GC content, because these processes allow for proportional changes in rates that apply to all sites, while genome reduction could involve an increased rate of loss in only a subset of genes (Blanc et al. 2007a). In other words, genome reduction might involve between-site heterogeneity in the extent of non-stationarity.

To see this, we simulated the evolution of the *Rickettsia* accessory genome over the topology of Figure 2a, using parameters estimated from the real data, and then estimated the correlation coefficient corresponding to the regression shown in Figure 3a. When data were simulated under a stationary model, we very rarely reproduced the correlation observed for the real data (Fig. 3b), nor could we reproduce the correlation when data were simulated under a standard non-stationary model (such that rates of gene gain and loss varied over lineages). However, if we combined the results of these simulations, and assumed that half of the genes evolved by the stationary process, and half by the non-stationary process, then we could produce a negative correlation (Fig. 3b—“Combined—random”). Furthermore, the correlation became strongly negative if we biased the sampling, such that the genes that were most often present evolved via the stationary process, and the genes that were most often absent evolved via the non-stationary process (Fig. 3b—“Combined—biased”). These data were all simulated over the topology of Figure 2a, and yet can lead to misleading phylogenetic placements, similar to those of Figure 2b, when their phylogeny is reconstructed using a single phylogenetic model (see, e.g., Supplementary Fig. S6).

Taken together, our analyses show strong support for a single underlying topology of the genus *Rickettsia.* Thirty-one out of 45 resolved nodes are supported by multiple source of phylogenetic signal (i.e., indels, rearrangements, or gene presence/absence, in addition to SNPs), and 18/45 by three or more (Fig. 2). Some placements do vary when the topology is estimated from the presence/absence of accessory genes (Fig. 2b), but we have argued that this is most likely to result from heterogeneity in the evolutionary process (between-site heterogeneity in non-stationarity) that is consistent with convergence on a minimal core genome and that is not accounted for by existing methods of phylogenetic inference.

### Testing for Recombination in the Rickettsia Core Genome

While support for the tree in Figure 2a is strong, it remains possible that this phylogeny is a consensus across the genome, but not the true tree for all—or even any—of the core genes (Beiko et al. 2008), and this possibility is supported by previous evidence of gene exchange events in *Rickettsia* (e.g., Blanc et al. 2007b; Gillespie et al. 2012, 2014a). To investigate this possibility, we tested for recombination in the core genes. We first compared individual core gene trees to the phylogeny of Figure 2a. In agreement with previous studies (Merhej et al. 2011; Hernandez-Lopez et al. 2013), several genes were congruent with the genome tree with strong support (see Supplementary Table S6 for a list), but we also found high levels of disagreement, with 62% (282/458) of gene trees containing at least one strongly supported (>95%) grouping contradicted with strong support in the genome tree (Supplementary Table S4). A multiple co-inertia analysis (de Vienne et al. 2012; Hernandez-Lopez et al. 2013) showed no strong tendency for disagreements to involve particular genes or strains (all strain outlier scores were all <0.25, nowhere near the recommended value of 0.5; de Vienne et al. 2012; Supplementary Fig. S5). However, the strains with the highest scores (R. symbiont of *A. bipunctata*; *R. felis*, *R. helvetica*, and R. symbiont of *I. scapularis*) were also misplaced in the accessory genome tree (Fig. 2b).

#### Simulations

These results could indicate widespread non-tree-like evolution in the core genome, but incongruity between gene tree topologies can also result from systematic error (Betancur-R. et al. 2013; Sharma et al. 2014). Suggestively, the most incongruent genes are more likely to be shorter than average (such that a few homoplasious sites could dominate the signal), and to have a wider variation of GC content among strains (which could lead to systematic error under a stationary model; Supplementary Fig. S7).

To ask whether systematic error might explain topological disagreements among gene trees, we simulated a complete set of core genes over a single phylogeny. Our simulation model included several sources of evolutionary heterogeneity, with parameters estimated directly from the real *Rickettsia* data. In particular, our simulations included (1) lineage-specific variation in base composition; (2) lineage-specific variation in genome-wide evolutionary rate; (3) gene- and site-specific variation in rates that applies to all lineages; (4) lineage-specific variation in the relative rate of third codon positions (due, e.g., to some lineages being under reduced selective constraint); and (5) gene-specific variation in the relative rate of third codon positions (due, e.g., to some genes being under reduced selective constraint in all lineages).

Trees estimated from our simulated core genes showed a high level of disagreement with the topology over which they were simulated. In particular, 27% (122/458) of the simulated core gene trees contained a well-supported disagreement. This level of incongruence is lower than that observed for the real genes (62% vs. 27%), but this is not particularly surprising, because our simulation model is almost certainly simpler than the real-world evolutionary process that generated the *Rickettsia* data (Thomas and Hahn 2015). For example, our simulations neglected non-independence between sites, and changes in relative rates that apply only to certain genes in certain lineages (i.e., gene-by-lineage effects); and both of these must be common in real-world molecular evolution (Williams and Hurst 2000; Smith and Eyre-Walker 2003; Breen et al. 2012).

**Figure 4.**
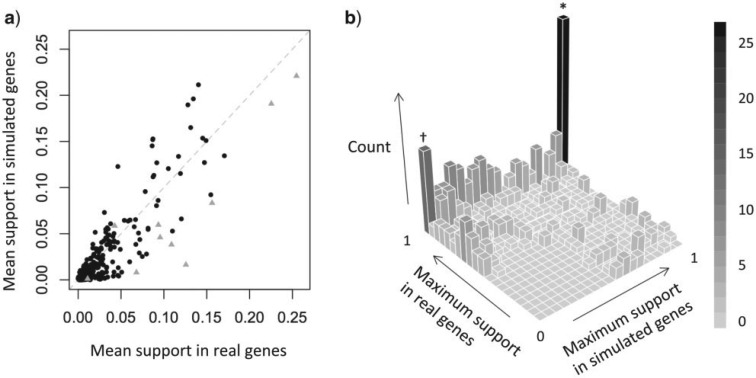
Support for nodes not found in the genome tree, from phylogenetic analysis of real core single genes and simulated core single genes (all of which were simulated over the genome tree). a) The mean posterior support for a placement across all genes (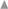 indicates the 10 putative gene exchange events identified by the synteny block analyses, with the two highest values involving the placement of the R. symbiont of *A. bipuncata* relative to *R. canadensis*). b) The maximum posterior support for a placement in any single gene. In both panels, only placements with >50% support in either the real or simulated data sets were included.

More tellingly, there is substantial overlap in the types of incongruence observed in the real and simulated data. First, the overall level of support for incongruent placements (not present in the core genome tree) is highly correlated between the real and simulated genes, i.e., incongruent placements that appear often in the real data also appear often in the simulated data (Fig. 4a, Supplementary Tables S4 and S5); and with no clear outliers, i.e., anomalous groupings that were regularly supported by the real genes, but not the simulated genes. Second, if we consider levels of support in single genes (since recombination might affect only very few genes, and therefore not be reflected in an average support over all genes), for incongruent placements observed with >95% support in at least one of the real genes, the most common maximum support among the simulated genes is also >95% (see the bin marked asterisk in Fig. 4b); i.e., for many anomalous placements, occasional strong support in the real genes is mirrored by occasional strong support in the simulated genes. Third, the multiple co-interia analyses yielded a comparable distribution of outlier scores (i.e., a few strains were equally impressive “outliers” in the fixed-topology simulations) (Supplementary Fig. S5a).

#### Incongruence between interspersed partitions

Despite the real similarities, there are also important differences between results for the simulated and real genes. For example, outlier scores for individual strains were only weakly correlated (Supplementary Fig. S5b). Moreover, some incongruent placements never received significant levels of support in the simulated genes, but were strongly—albeit rarely—supported in the real genes. In fact, there are 18 such nodes, found in 15 genes (see bin marked dagger in Fig. 4b; Supplementary Table S7). These disagreements are the best candidate instances of gene exchange in the core genome, although they too might represent systematic error that was not captured by our simulation procedure.

To further examine these 18 incongruent placements, we re-estimated the gene trees to test for evidence of “internal contradictions” (i.e., strongly conflicting phylogenetic signal from interspersed sets of sites that must, in reality, have followed the same evolutionary history). Half of the incongruent groupings (9/18), which were the strongest candidates for gene exchange, showed evidence of internal contradictions (i.e., support for contradictory topologies from completely interspersed sets of sites, without any evidence of intra-gene recombination). A significant minority of nodes (8/18), showed no internal contradictions, but further examination suggested than none was a convincing recombinant. In particular, 2/18 contained 1 bp indels toward gene boundaries, and these single events were erroneously counted as multiple SNPs, while other true SNPs supported Figure 2a (Supplementary Fig. S8). The remaining 6/18 disagreements involve unusual placements of multiple basal nodes, and so could not have been generated by a single gene exchange event, and all 6 included high rates of homoplasy over their favored topology, with multiple SNPs contradicting this topology and supporting Figure 2a (see Supplementary Table S9 for details).

Internal contradictions provide strong evidence of systematic error, but the approach cannot be applied to single genes whose protein sequence is conserved. As such, we applied the approach to 55 “synteny blocks” of core genes, which maintained their relative order over most of the *Rickettsia* phylogeny. As with the single core genes, initial analyses of the synteny blocks suggested widespread gene exchange. Indeed, for 46/55 blocks, at least one partition yielded a highly supported placement that disagrees with Figure 2a (Supplementary Table S8). However, 21 of these blocks showed strong internal contradictions, strongly suggestive of systematic error, while only 15 had incongruent placements that were consistently supported. Furthermore, these two sets of incongruous placements are remarkably similar: 13/15 consistently supported placements are involved in strong internal contradictions on other blocks, and placements that are more commonly (i.e., by more blocks) supported across sets of interspersed sites are also more commonly contradicted across sets of interspersed sites (Kendall's rank test, P=0.02). Moreover, the 15 misplacements were inferred with similar frequencies in our simulated and real single-gene trees (gray triangles in Fig. 4a), 9/15 were inferred with strong support in our simulated gene trees (contributing to the bin labeled with an asterisk in Fig. 4b), and none are supported by indels in the same genes.

#### Gene exchange or systematic error?

**Figure 5.**
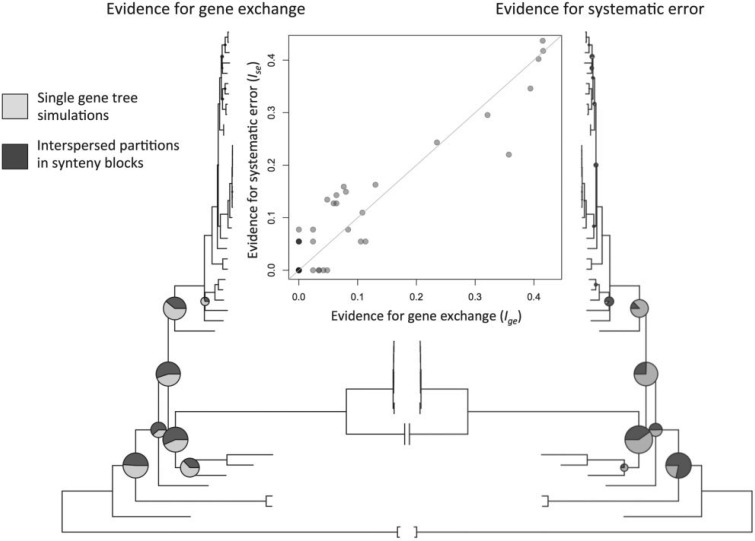
High levels of putative gene exchange occur at nodes that have high levels of systematic error. The size of the pie charts corresponds to the combined evidence for gene exchange ($I_{ge}$; left-hand phylogeny), or systematic error ($I_{se}$; right-hand phylogeny). The plot shows the correlation between these two quantities. The darker portions of the pie charts show much of the evidence comes from analysis of real or simulated, single-gene trees ($p_{ge}$ and $p_{se})$, as opposed to analyses of interspersed sites in synteny blocks ($q_{ge}$ and $q_{se})$ (see “Methods” section for full details).

We combined the analyses above to compare possible instances of gene exchange, and observed levels of systematic error, across the *Rickettsia* core genome tree. In particular, we defined two indices. The first, $I_{ge}$, measures the extent to which a given node in the genome tree is contradicted by (1) analyses of single core genes, and (2) analyses of synteny blocks without evidence of internal contradiction. The second, $I_{se}$, is the same quantity for *simulated* core genes (all of which were simulated over the genome tree), and placements in the synteny block trees that do show strong internal contradictions. High values of $I_{ge}$ might suggest that the presence of the node is in doubt due to frequent gene exchange, but high values of $I_{se}$ must indicate that the presence of the node varies due to systematic error. Figure 5 shows that $I_{ge}$ and $I_{se}$ are strongly correlated (see also Supplementary Fig. S9). As such, the putative gene exchange events involve placements that are most prone to systematic error. In addition, both values are highest for deep nodes, where we would expect greater problems inferring the phylogeny. This makes it difficult to reject the hypothesis that all or most of the putative gene exchange in the core genome is, in fact, due to systematic error.

### Presence of Conjugation Genes

The hypothesis of gene exchange in the *Rickettsia* core genome would be strengthened if the strains with the least stable placements were shown to undergo higher levels of gene exchange elsewhere in the genome. A direct approach to answering this question would be to examine gene trees of individual accessory genes, but—no less than the core gene trees—these might be subject to high levels of systematic error. An alternative, indirect approach is to examine between-strain variation in the machinery required for gene exchange. Fourteen out of 51 of our *Rickettsia* genomes contain one or more complete conjugation genes, either chromosomally or on plasmids (Supplementary Table S10; see also Gillespie et al. 2007, 2012, 2014a, 2014b; Weinert et al. 2009b), and the presence of these genes appears unusually variable across the tree (e.g., one of the *R. massiliae* strains has 13 conjugation genes on its chromosome, and the other has none; Blanc et al. 2007b). Furthermore, genomes with more conjugation genes are significantly larger, after correcting for relatedness (GLS regression of chromosome size against number of conjugation genes, $P<10^{\text{-5}}$). This may be a passive correlation, but it is at least consistent with the hypothesis that conjugation genes are involved in gene gain. However, most importantly for the present study, the presence of conjugation genes shows no correlation with levels of phylogenetic incongruence (number of conjugation genes does not predict multiple co-inertia analysis strain scores; GLS regression, P=0.17). This test is highly indirect, and other indicators of gene exchange must have been missed (not least any conjugation genes on plasmids that were lost during passage; Baldridge et al. 2008). Nevertheless, we find no evidence to contradict our conclusion that the phylogenetic incongruence we observed is due to systematic error.

## Discussion

We have estimated the phylogeny of the *Rickettsia* genus using SNPs, indels, and re-arrangements in the core genome. We have shown that there is strong support for a single underlying topology for the core genome (Figs. 1 and 2a), which accounts for most of the data, and is generally consistent with previous studies (e.g. Gillespie et al. 2007, 2008, 2014a; Vitorino et al. 2007; Merhej et al. 2009a, 2014; Weinert et al. 2009a; Merhej and Raoult 2011; Driscoll et al. 2013; Weinert 2015).

We have shown that a similar topology is inferred from gene presence/absence, and have argued that the remaining disagreements are plausibly attributed to convergent patterns of gene loss, toward a common minimal core genome (McCutcheon and Moran 2012). In terms of phylogenetic inference, this corresponds to a non-stationary process that applies to an unknown subset of sites (or, equivalently, variation in the degree of non-stationarity across sites), and thus cannot be modeled by existing methods. Such heterogeneity in the process of gene gain/loss could be widespread, for example, an artifactual clustering of genomes of similar size has been observed in the eukaryotic tree (Lake and Rivera 2004) and it will also have implications outside of phylogenetic inference, complicating, for example, the inference of ancestral gene content (Tuller et al. 2010).

After reconstructing the core genome tree, we went on to show extensive evidence of phylogenetic incongruence when the phylogeny is estimated from a small proportion of the core genome. These disagreements are widespread, but most involve the placements of a few strains (or equivalently a few basal nodes), namely the R. symbiont of *A. bipunctata* (newly sequenced here), *R. felis*, *R. helvetica* and R. symbiont of *I. scapularis* (Fig. 5 and Supplementary Fig. S5b), most of which are also misplaced in the accessory genome tree (Fig. 2b). This could be interpreted as evidence of widespread gene exchange, affecting both accessory and core genes (as previously described in Merhej et al. 2011; Hernandez-Lopez et al. 2013), but we have argued that most, and perhaps all, of these disagreements stem from systematic error in phylogenetic reconstruction. This argument was supported by several lines of evidence. These include (1) simulations over a single topology—which produced many of the same incongruent placements (Figs. 4 and 5); (2) contradictions between interspersed subsets of sites—which must be due to estimation error, and again involved very similar misplacements (Fig. 5 and Supplementary Fig. S9); (3) generally high levels of homoplasy in some putative recombinants (Supplementary Table S9); and—indirectly—(4) the lack of correlation between the presence of conjugation genes and levels of phylogenetic incongruence.

Furthermore, the vast majority of the putative gene exchange events occur where systematic error will be most severe: in genes that are smaller and with higher variation in GC content (Supplementary Fig. S7), and in lineages that are basal, and found on sparsely sampled parts of the tree (Fig. 5). Indeed, we never observe a strongly supported case of gene exchange that causes movement into or out of a densely sampled group (Supplementary Tables S4 and S8).

Moreover, the least stable placements have other properties that might make them more subject to systematic error. This is trivially true for the R. symbiont of *A. bipunctata*, which is poorly assembled. More interestingly, features of *Rickettsia* evolution might lead to greater instability in the placements of *R. helvetica* and the R. symbiont of *I. scapularis*. It is notable that hard tick (Ixodidae) hosts are found across the phylogeny (Fig. 1), and this is a plausible ancestral host state for the arthropod-associated species. Lineages that have evolved a new ecology, involving different host associations, are sometimes associated with faster evolutionary rates (particularly in amino acid changing sites, and in accessory gene content). This is clearest for the Typhus group, which infects lice and fleas, but is also true of *R. akari*, which infects mites (Fig. 1). *R. helvetica* and the R. symbiont of *I. scapularis* are placed on either side of a clade that includes these divergent strains. Furthermore, both share the putative ancestral host range, and have similar GC contents, gene contents, and short root-to-tip distances (Fig. 1). Their genomes are not extreme, but due to their close relatives, these shared (likely ancestral) states could lead to artificial attraction in a phylogenetic reconstruction (as found in, e.g., Fig. 2b and Supplementary Table S8; Philippe et al. 2005; Zhong et al. 2011). Of course, the shared hosts might also provide opportunity for gene exchange, but this host range is also shared with the well-sampled Spotted fever group, whose placements remain quite stable (Fig. 5).

It is important to stress that all of the results above remain consistent with gene gain (the uptake of foreign DNA from within or outside of the genus) playing an important role in *Rickettsia* evolution (Blanc et al. 2007b; Gillespie et al. 2007, 2012, 2014a; Weinert et al. 2009b; Merhej et al. 2011; Hernandez-Lopez et al. 2013), and do not undermine previous studies that have convincingly demonstrated specific instances of horizontral gene transfer, such as those found on the pLbaR plasmid of a recently sequenced *R. felis* genome and those present in the integrative conjugative elements of the R. symbiont of *I. scapularis* (Gillespie et al. 2012, 2014a). Furthermore, some gene exchange events are difficult to reconstruct in principle (e.g., recombination between sister lineages or conserved sequences). Nevertheless, we do conclude that there is little compelling evidence for non-tree-like evolution in the *Rickettsia* core genes, or in the general process of gene gain and loss in the accessory genome.

Our results also have implications for the study of reticulate evolution in general. There has been extensive debate over whether the evolution of genomes, particularly bacterial, is best described by a single tree, or by a network of gene transfer events (e.g. Doolittle 1999; Kurland et al. 2003; Lerat et al. 2003; Koonin 2009; Raoult 2010), and such reticulate evolution has even been described as “non-Darwinian” (unhappily, since Darwin was well aware of hybridization). Phylogenetic incongruence between individual gene trees and the species tree has been rightly described as the “gold standard” for identifying horizontal transfer (Keeling and Palmer 2008), but our analysis confirms that this signal cannot always be relied upon. Even with an analysis restricted to a single bacterial genus, we have shown high levels of false positives, stemming from changes in the evolutionary process that are not easily captured by phylogenetic models. Similar caveats must apply to studies testing for horizontal gene transfer across distantly related organisms, particularly when considering deep nodes or sparsely sampled lineages of a phylogeny, and therefore especially across different domains of life (Gladyshev et al. 2008; Puigbò et al. 2009; e.g. Boschetti et al. 2012; Crisp et al. 2015).

## Supplementary Material

Data available from the Dryad Digital Repository: http://dx.doi.org/10.5061/dryad.6cn66.
